# Family composition and living arrangements—Cross‐sectional study on family involvement to self‐managed rehabilitation of people with coronary artery disease

**DOI:** 10.1002/nop2.555

**Published:** 2020-07-02

**Authors:** Sonja Tuomisto, Meeri Koivula, Päivi Åstedt‐Kurki, Mika Helminen

**Affiliations:** ^1^ Faculty of Social Sciences Health Sciences University of Tampere Tampere Finland; ^2^ Pirkanmaa Hospital District Tampere Finland; ^3^ Research, Development and Innovation Centre Tampere University Hospital Tampere Finland

**Keywords:** coronary artery disease, family, family members, involvement, living arrangements, rehabilitation

## Abstract

**Aim:**

To describe the family composition and living arrangements of persons diagnosed with coronary artery disease and those relationships to family involvement in self‐managed rehabilitation.

**Design:**

A cross‐sectional study.

**Methods:**

Data were collected with postal questionnaire from persons diagnosed with coronary artery disease (CAD) by using the Family Involvement in Rehabilitation (FIRE) scale. It measures family members' promotion of patients' rehabilitation and issues encumbering rehabilitation in family. Statistical methods were used to analyse the data.

**Results:**

Patients' gender and having children in the family were predictors of issues encumbering rehabilitation in the family. But when examining living arrangements, patients who lived with a spouse or underage children had a better environment for recovery than those who lived alone or with adult children. More attention should be paid to targeting appropriate support for persons with coronary artery disease and their family members during the rehabilitation phase.

## 1 INTRODUCTION

It has been extensively acknowledged that family relationships and the progress and treatment of illnesses have a connection. Several assessment tools and interventions have been developed to advance family health and healing (Årestedt, Persson, & Benzein, 2014; Bell, 2009; Wright & Leahey, 2013). To understand the meaning of family in the life of a person with a severe illness, we need to define what a family is, the concept of which is far‐reaching and subjectively defined. Family can include various people besides those with whom we are connected by biological or legal ties; for some, emotional ties or concrete support have greater significance. How we define the concept of family has changed over time. Family compositions have become more diverse, and the traditional nuclear family is less common. In the last century, there have been considerable changes in family structure in Western societies. This was partially a consequence of the growing number of divorces and reconstituted families. (Chambers, 2012; Roberto & Blieszner, 2015) It is also notable that family members are not necessarily the people who live in the same household. Currently, older people often live on their own or with a spouse. In the mid‐twentieth century, it was very uncommon to live alone in old age (Chambers, 2012). In this study, the persons with coronary artery disease themselves define who belongs in their family.

In the health sciences, family involvement has been examined in various contexts: mental health care (Kontio, Lantta, Anttila, Kauppi, & Välimäki, 2017), the care of older people (Palonen, Kaunonen, & Åstedt‐Kurki, 2016), decision‐making (Itzhaki, Hildesheimer, Barnoy, & Katz, 2016) and from the perspective of healthcare professionals (Luttik et al., 2017). The most crucial thing is to define the concept of involvement because it can have many different meanings and manifestations. In this study, family involvement refers to how family members engage in the rehabilitation of a person diagnosed with coronary artery disease (CAD) and involvement is seen from two different viewpoints: family promoting rehabilitation and issues encumbering rehabilitation in family, which derive from the previous literature (Benyamini, Medalion, & Garfinkel, 2007; Cartledge, Feldman, Bray, Stub, & Finn, 2018; Dalteg, Benzein, Fridlund, & Malm, 2011; Hansen, Zinckernagel, Schneekloth, Zwisler, & Holmberg, 2017; Jackson, McKinstry, Gregory, & Amos, 2012; Kärner, Dahlgren, & Bergdahl, 2004; Mahrer‐Imhof, Hoffmann, & Froelicher, 2007; Rantanen et al., 2008; Wong et al., 2016). It has been shown that, in addition to spouses, children are special supporters for patients with CAD (Roos, Rantanen, & Koivula, 2012), but studies of the significance of other family members living in the same household are scarce. Living arrangements have been found to be strong determinants for survival after myocardial infarction. Heart attack risk is greater for those who live alone or those who are not married, no matter the person's age (Kilpi, Konttinen, Silventoinen, & Martikainen, 2015; Lammintausta et al., 2014).

CAD is a lifelong illness that a person has to adapt to living with. Cardiac rehabilitation contains three important parts: guidance for training and physical activity, heart‐healthy lifestyle and counselling to reduce stress (American Heart Association, 2017). In this study, rehabilitation refers to the patient's self‐managed rehabilitation based on the guidance offered during the hospital stay. Thus, the patient education offered by the hospital enables self‐managed rehabilitation. The importance of communication between the healthcare professionals and the person with heart disease is particularly emphasized in the hospital discharge phase and in the rehabilitation phase encounters, which are critical points for conveying the necessary information. This can promote the person's ability to self‐care and prevent the recurrence of the disease. (Chew et al., 2016)

When a family is confronted with an acute or chronic cardiac event, the role of family and caregiver involvement in education is pivotal. In the event of a serious illness, family members often must adopt new kinds of responsibilities concerning the role of a caregiver, such as observing symptoms or support in uptaking healthy lifestyle (Commodore‐Mensah & Dennison Himmelfarb, 2012). Overprotection, communication problems, challenges in daily routines and adjustment to illness are examples of potential difficulties (Dalteg et al., 2011). Guidance and self‐care support should be offered to not only the patients but also to their families, which is an important way of enhancing rehabilitation at home and reducing hospital readmissions. Patients' follow‐up care should be carefully planned with the patient and with family members. These considerations should be incorporated into postevent rehabilitation. (Cebolla & Bjornberg, 2017).

It is often assumed that the family is helpful and supportive during the rehabilitation process, but more information is needed from the patient's perspective to better address possible family life challenges. To develop patient education in the rehabilitation phase, it is essential to gain new knowledge about patients' self‐managed rehabilitation at home among family members. This knowledge is essential, for example, for delivering client‐ and family‐centred care, promoting self‐management and providing client and caregiver education (Vaughn et al., 2016).

Information searches were conducted on this topic originally in 2012 and 2013, and the searches were updated in spring 2019. Databases that were used were as follows: CINAHL, Medline, Medic, Cochrane library, Medic and PsycInfo. All together titles/keywords of 986 studies were read through and after that 64 abstracts. Manual searches have also been used to find the latest research on the subject. Very few studies concerning this topic (e.g. Cartledge et al., 2018; Hansen et al., 2017; Kähkönen, Kankkunen, Miettinen, Lamidi, & Saaranen, 2017; Köhler, Nilsson, Jaarsma, & Tingström, 2017) have been published in the last 3 years, which makes this article important and strengthens the novelty of it.

The aim of this paper was to describe the family composition and living arrangements of persons diagnosed with CAD and their connections to family involvement in rehabilitation. The research questions were as follows:

1. What is the family composition and what are the living arrangements of persons diagnosed with CAD?
2. How are family composition and living arrangements associated with family involvement in the rehabilitation of persons with CAD?

## 2 METHODS

### 2.1 Design

Convenience sampling was used in this descriptive cross‐sectional study, which was carried out in one university hospital in Southern Finland. This study is third part of a larger research project with pilot study (Tuomisto, Koivula, & Joronen, 2014) and earlier publication (Tuomisto, Koivula, Åstedt‐Kurki, & Helminen, 2018) based on the same empirical data.

### 2.2 Participants

The inclusion criteria were as follows: patients diagnosed with coronary artery disease who had been undergoing hospital treatment (inpatient) and who assented to participate in the study. The onset of the disease or treatment received by the individual was not limited in any way. Patients who underwent angiography, thrombolytic therapy, percutaneous coronary intervention (PCI) or coronary artery bypass grafting (CABG) participated in the study. The exclusion criteria were as follows: patients who did not speak Finnish or who, for some reason (poor health condition, poor vision or serious mental health problems), were not capable of answering the questionnaire. Power analysis was used in this study to determine suitable sample size. The results from a pilot study (Tuomisto et al., 2014) were used. The standard deviation of 0.7 was used in the computation (calculated from the Family promoting rehabilitation and Issues encumbering rehabilitation in family subscales) and the mean sum score value with 95% confidence interval and a marginal error no more than 0.1. According to this, the suitable sample size is 189 respondents (Levy & Lemeshow, 1991). The final sample size estimate is 218, when taking into consideration the non‐response rate of 15% in the preliminary study. The total number of returned questionnaires was 172, and three questionnaires were rejected because of missing answers. It was considered in collaboration with statistician that although the response rate (79%) was lower than in the pilot study, the number of returned questionnaires (*N* = 169) was enough for this study.

### 2.3 Data collection

Patients were recruited from an information group for patients with CAD and additionally from cardiac wards. The hospital where the data were collected arranges an information group for patients diagnosed with coronary artery disease. The patients were invited to this group during their hospital stay. The information group is organized every 5 weeks and the meeting includes lectures by various experts on coronary heart disease and its treatment. The intent is for the patient to attend only one briefing and family members may also attend. At the end of the information group, the patients got information related to this research and they gave written informed consent. Postal questionnaires were mailed to them at least 6 weeks after discharge from the hospital. This time frame allowed the patients to spend time at home with family members after leaving the hospital. The patient filled in the questionnaire at home and returned it with free postage. The data collection was conducted between May 2013–July 2015.

### 2.4 Instruments

In 2014, we developed the Family Involvement in Rehabilitation (FIRE) scale for this study and testing of the scale was done during this four part research project and the results of internal and concurrent validity and reliability of the subscales have been published already on earlier papers (Tuomisto et al., 2014, 2018). The content of FIRE is based on a literature review, and it measures patients' perceptions of family involvement in the rehabilitation of persons with CAD. The scale has two parts: *Family promoting rehabilitation* (16 items) and *Issues encumbering rehabilitation in the family* (30 items). A 6‐point Likert scale was used (1 = strongly disagree; 2 = disagree; 3 = slightly disagree; 4 = slightly agree; 5 = agree; 6 = strongly agree). The structure of the scale and the items is presented in Table 1. The questionnaire also contains 17 questions concerning demographic characteristics, such as age, gender, family members, living arrangements, family relations and the history of CAD and its treatment (Table 2).

**TABLE 1:** The items and the structure of the Family Involvement in Rehabilitation (FIRE) scale

Family promoting rehabilitation (16 items)
Enabling good circumstances (4 items)
1. My family helps me with daily chores
2. My family members try to protect me from additional stress
3. My family is sympathetic to my illness
4. My family acts in agreement with me
Family closeness (4 items)
5. My family takes care of me
6. Having a family makes my recovery easier
7. The presence of family members makes me happy
8. My family keeps in touch with me
A family member as a carer (4 items)
9. My family seeks information about my illness
10. My family supports me with issues concerning my care
11. My family members support me in treatment‐related decision‐making
12. My family observes symptoms of my illness
Motivating patient (4 items)
13. My family's attitude towards my illness discourages me
14. It is impossible to discuss different options with my family
15. My family members have a positive attitude towards my recovery
16. My family members support me in lifestyle changes

Issues encumbering rehabilitation in family (30 items)
Future uncertainty (4 items)
1. Poorly planned treatment causes uncertainty for me and my family members
2. Uncertainty about the future makes it difficult to commit to lifestyle changes
3. Lifestyle changes cause negative reactions in our family
4. My family has had to adjust to the sudden changes in my health
Inadequate support from nursing staff (4 items)
5. Support from healthcare staff is deficient
6. Insufficient support from nursing staff causes stress to my family members
7. Informational support for my family members is inadequate
8. My family members do not have enough information about what is good for me
Processing emotions (4 items)
9. My illness causes me fear and anxiety
10. I feel like I am losing my temper more easily than before
11. My illness causes anxiety and fear for my family members
12. We cannot express the feelings that my illness has caused with family members
Family's coping with everyday life (9 items)
13. Performing daily responsibilities worries me
14. Performing daily responsibilities worries my family
15. Sharing everyday responsibilities causes stress in our family
16. My family's financial situation worries me
17. My illness causes changes to family life
18. I am concerned about my family's coping during my rehabilitation
27. I feel stressed when I ask for help from my family
28. I wish my family wouldn't worry so much about my illness
30. My family members do not support me enough in my rehabilitation
Family interaction (5 items)
19. Misunderstandings cause trouble between family members
20. The difficulty talking about things causes problems in our family
21. Different expectations cause problems between family members
22. There have been problems in my sex life since I became ill
29. I want to protect my family from concerns by hiding some issues related to my illness
Family responsibilities for the patient (4 items)
23. Excessive caring of family members annoys me
24. Taking responsibility for my rehabilitation is a concern for my family
25. My illness has limited the life of other family members
26. My family members' personal time has decreased because of my illness

**TABLE 2:** Demographic characteristics, information related to CAD, family composition and living arrangements of persons diagnosed with CAD

Demographic characteristics	*N*	%
Gender		
Male	129	76
Female	40	24
Age		
60 years or less	40	24
61–74 years	93	55
75 years or more	36	21

Information related to CAD		
Onset of symptoms		
≥10 years ago	34	20
4–9 years ago	45	27
≤3 years ago	82	48
Missing	8	5
Appearance of heart symptoms		
Not even at exertion	56	33
With minor exertion	39	23
With heavy exertion	43	25
Also at rest	25	15
Missing	6	4
Earlier chest pain treatments of CAD in hospital	103	61
Myocardial infarction	72	43
Thrombolytic therapy	22	13
Angiography	163	96
PCI^a^	126	75
CABG^a^	18	11

Family members	*N* yes	*N* no	% yes	% no
Spouse	151	18	89	11
Own children	75	94	44	56
Spouses' children	8	161	5	95
Sister/Brother	9	160	5	95
Other relative	2	167	1	99
Friend	4	165	2	98
Colleague	0	169	0	100
Somebody else	2	167	1	99

Living in the same household	*N*	%
Alone	14	8
With spouse	131	77
With children under 18 years old	11	7
With grown children	11	7
With somebody else	2	1

^a^ CABG, coronary artery bypass grafting; CAD, coronary artery disease; PCI, percutaneous coronary intervention.

A pilot study (*N* = 29), which purpose was to test the scale before conducting the actual research with larger data, demonstrated that the questions were understandable to patients. It also gave information about concurrent validity, which was fairly good. Cronbach's alpha coefficient was used to evaluate the reliability of the FIRE scale, and the values of the subscales ranged 0.502–0.928 (Tuomisto et al., 2014). The content validity of the FIRE scale, which consist of Family promoting rehabilitation (Hagan, Botti, & Watts, 2007; Kärner et al., 2004; Mahrer‐Imhof et al., 2007; Stewart, Davidson, Meade, Hirth, & Makrides, 2000) and Issues encumbering rehabilitation in family (Benyamini et al., 2007; Dalteg et al., 2011; Kärner et al., 2004; Rantanen et al., 2008), is based on several earlier studies.

On these bigger data, Cronbach's alpha coefficient values can be interpreted as good (0.681–0.933 value range). The Cronbach's alpha coefficient for the family promoting rehabilitation part was 0.933, and for issues encumbering rehabilitation part 0.930. More detailed information about the reliability and validity of the FIRE scale has been considered in the previous article (Tuomisto et al., 2018).

### 2.5 Ethics

A positive statement was obtained from the hospital ethics committee (The Regional Ethics Committee of University Hospital, approval number R13018H) and the administrators at the clinic granted permission to carry out the study. Informed consent was requested from the patients when they received verbal and written information about the study. Patients were notified that they had the right to refuse to take part and that they could discontinue participation at any time. Information about the confidentiality of personal data was also declared. The signed consents and questionnaires were coded in case they ever needed to resubmit the questionnaire. It was ensured that the anonymity of the respondents remained throughout the study (World Medical Association, 2017).

### 2.6 Data analysis

Data were analysed using IBM SPSS (Statistical Package for Social Sciences) Statistics for Windows, version 22 (IBM Corp., Armonk, NY, USA). Demographic characteristics are presented using frequencies and percentages. To describe the data, means and standard deviations are given for normally distributed variables and medians and quartiles (Tukey's Hinges) for subscales with a skewed distribution. A binary logistic regression analysis was used to examine the connections between family composition and family involvement (family promoting rehabilitation and issues encumbering rehabilitation), where the values of the two parts were dichotomized to higher or lower than the median/mean. This was done because the FIRE scale was developed for this study and the exact limits for good or acceptable family involvement were not yet specified. However, when choosing the median (or mean when normality assumption is met) as a cut‐point, there are an equal number of cases in both groups, enabling solid model estimates and this cut‐point more or less identifies the highest or lowest (the best or the worst, depending on which scale is used) involvement scores. High family involvement was used as a dependent variable, with age, gender and family members as independent variables. These additional background factors were chosen for the model because they have been found to associate with different types of challenges during cardiac patients' rehabilitation (Ghezeljeh et al., 2010; Koivula, Hautamäki‐Lamminen, & Åstedt‐Kurki, 2010; Ky et al., 2010). The meaning of children living in the family was further explored by examining the connections between living arrangements and the subscales of the two parts: family promoting rehabilitation and issues encumbering rehabilitation in the family. The groups were compared using one‐way ANOVA and Kruskal–Wallis tests. A *p‐*value of <.05 is considered to be statistically significant (Munro, 2005).

The FIRE questionnaire asked about family structure in this way: *Which of the following persons belong to your family*? The respondent was able to choose from several alternatives (Table 2). To perform the logistic regression analysis, the family structure was recategorized into three groups: spouse, children in the family and other family members. This recategorization does not exclude other answers so that those who include a spouse can also name children or other family members.

## 3 RESULTS

In total, 172 questionnaires were returned and the response rate was 79%. Three questionnaires were rejected because of a substantial number of missing answers.

### 3.1 Descriptive statistics of the sample

Most participants were men (76%), and the average age was 67 years. Approximately half had been diagnosed with CAD within 3 years (49%), others had had CAD at least 4 years. The preponderance of respondents (61%) had been treated in hospital from one to twenty times because of chest pain. The average number of hospital treatments was two. Other disease‐ and treatment‐related information can be found in Table 2.

Most of the respondents reported a spouse (89%) as a family member. Almost half (44%) of the participants perceived their own children as family members and 5% considered their spouses' children as such. A few respondents (5%) reported brothers or sisters as part of the family. The preponderance of participants lived in the same household as a spouse (77%) and 8% lived alone (Table 2).

### 3.2 Living arrangements and family involvement

Living arrangements were associated with enabling good circumstances insofar as patients who lived with a spouse or underage children had better circumstances for recovery than those who lived alone (Table 3). Living arrangements were not significantly associated with other subscales of family promoting rehabilitation.

**TABLE 3:** Living arrangements and family involvement

Background variable	*N*	Family promoting rehabilitation							
		Enabling good circumstances		Family closeness		Family member as a carer		Motivating patient	
		Md (Q1/Q3)	*p*	Md (Q1/Q3)	*p*	Md (Q1/Q3)	*p*	Md (Q1/Q3)	*p*
Living in the household			**.008**		.134		.304		.275
(1) Alone	14	18.0 (15.0/19.0)		19.5 (17.0/23.0)		18.5 (15.0/22.0)		22.0 (20.0/24.0)	
(2) With spouse	131	20.0 (18.0/23.0) 1 < 2***		22.0 (20.0/24.0)		21.0 (19.0/23.0)		21.0 (19.0/23.0)	
(3) With spouse and children under 18 years old	11	20.0 (18.0/21.0) 1 < 3*		21.0 (20.0/23.0)		20.0 (19.0/21.5)		20.0 (17.5/20.0)	
(4) With spouse and grown children	11	19.0 (17.0/21.0)		22.0 (19.0/23.5)		19.0 (16.5/23.0)		20.0 (16.0/23.5)	

Background variable	*N*	Issues encumbering rehabilitation in family											
		Future uncertainty Md (Q1/Q3)	*p*	Inadequate support from nursing staff Md (Q1/Q3)	*p*	Processing emotions *M* (*SD*)	*p*	Family's coping with everyday life *M* (*SD*)	*p*	Family interaction Md (Q1/Q3)	*p*	Family responsibilities for the patient Md (Q1/Q3)	*p*
Living in the household			**.018**		.771		**.011**		**.004**		**.013**		**.001**
(1) Alone	14	9.0 (8.0/12.0) 1 < 4*		8.0 (7.0/10.0)		10.0 (4.4) 1 < 4**		22.0 (8.1) 1 < 4**		11.0 (5.5/15.0) 1 < 4*		6.0 (4.0/10.0) 1 < 4**	
(2) With spouse	131	11.0 (9.0/14.0)		8.5 (7.0/13.0)		12.4 (3.8)		25.5 (7.3)		12.0 (10.0/16.0)		10.0 (8.0/13.0) 1 < 2*	
(3) With spouse and children under 18 years old	11	8.0 (7.5/10.0)		9.0 (8.0/12.5)		13.0 (3.3)		25.1 (8.8)		14.0 (8.5/17.0)		8.0 (6.0/10.0)	
(4) With spouse and grown children	11	14.0 (10.5/15.5)		10.0 (7.5/14.5)		15.1 (3.2)		32.9 (7.5)		20.0 (13.5/22.0)		11.0 (10.0/14.5)	

Abbreviations: *M*, mean; Md, median; Q1, lower quartile; Q3, upper quartile; *SD*, standard deviation.

* *p* < .05.

** *p* < .01.

*** *p* < .001 (*p*‐values < .05 are bolded).

Living arrangements had a strong connection with issues encumbering rehabilitation in the family: those who lived alone had the smallest values in all issues encumbering rehabilitation except future uncertainty. The most encumbering issues were perceived by those who lived with grown children (Table 3).

### 3.3 Family composition and family involvement

The relationship of family composition to family involvement was examined with a binary logistic regression analysis (Table 4). No statistically significant predictors of family promoting rehabilitation were found in this model, but the most powerful predictor of issues encumbering rehabilitation was the patient's gender. Men were more likely than women to have challenges (OR 2.6, *p* = .023). Having children in the family was also a predictor of issues encumbering rehabilitation (OR 2.3, *p* = .034).

**TABLE 4:** Predictors of family involvement in the rehabilitation process of a person with CAD (logistic regression analysis)

Variable	Family Involvement									
	*R* ^2^ ^a^	Family promoting rehabilitation^b^				*R* ^2^ ^a^	Issues encumbering rehabilitation^b^			
		*p*‐value	OR	CI 95%			*p*‐value	OR	CI 95%	
Age	0.009					0.004				
≤60		.583					.212			
61–74		.671	1.19	0.539	2.61		.194	1.7	0.756	3.97
≥75		.612	0.773	0.285	2.09		.136	2.2	0.779	6.23
Gender^c^	0.000	.739	0.878	0.410	1.88	0.034	.**023**	2.6	1.15	6.08
Family members										
Spouse^d^	0.003	.195	2.23	0.663	7.47	0.005	.249	2.0	0.607	6.83
Children in the family^d^	0.001	.570	0.818	0.409	1.64	0.013	**.034**	2.3	1.07	4.89
Other family members^d^	0.014	.449	1.56	0.493	4.94	0.001	.909	1.1	0.317	3.63

Note

*p*‐values < .05 are bolded.

Abbreviations: CI, confidence interval; OR, odds ratio.

^a^ Nagelkerke.

^b^ Model pursues to explain values higher than mean/median.

^c^ Male = 1, female = 0.

^d^ Yes = 1, no = 0.

## 4 DISCUSSION

This paper aims to describe the family composition and living arrangements of persons diagnosed with CAD, and how these factors relate to family involvement, which consists of family promoting rehabilitation and issues encumbering rehabilitation in the family. About family composition in this study, most of the respondents reported that they had a spouse and most also stated that they live with their spouse in the same household. In many previous studies concerning CAD patients and their family members, attention is often focused on the spouse (Cartledge et al., 2018; Eriksson, Asplund, & Svedlund, 2010; Franks et al., 2006; Köhler et al., 2017) and the roles of other family members have not widely studied. Although the spouse is the closest family member for the most part, it is essential to recognize the diversity of families and consider the roles of all family members during rehabilitation (Andersson, Borglin, Sjostrom‐Strand, & Willman, 2013; Roos et al., 2012).

In this study, family composition had no effect on how persons with CAD perceived family members' to promote their rehabilitation. Instead, living arrangements were associated with how family enables good circumstances for recovery. Living with a spouse or underage children seems to be beneficial. Earlier studies show parallel results; the advantages of a marital relationship may protect from myocardial infarction fatality and marriage also seems to be protective against out‐of‐hospital acute coronary syndrome (ACS) death (Gerward, Tydén, Engström, & Hedblad, 2010; Kilpi et al., 2015; Lammintausta et al., 2014). Persons with CAD who are married or live in cohabitation are also more likely to receive emotional support from their families than those without a partnership (Kähkönen et al., 2017). Living alone has also been found to relate to non‐attendance of cardiac rehabilitation programmes (Nielsen, Faergeman, Foldspang, & Larsen, 2008).

Those who live with a spouse are more likely to get help more easily, for example, for practical matters such as burdensome household chores. In this study, most respondents were over 60 years of age; their children are teenagers or older and can be very helpful, but they also need information about CAD to understand the nature of their parent's illness. On the other hand, living arrangements did not have any relation to other subscales of family promoting rehabilitation, which indicates that patients feel that family members can be supportive, regardless of whether they live at the same address.

In this study, gender was significantly associated with issues encumbering rehabilitation; for example, men seem to be more susceptible to different challenges in family relations. Previous studies have acknowledged gender differences in emotional expressiveness and recognition of emotions (Fischer & LaFrance, 2015; McKeown, Sneddon, & Curran, 2015); women are found to be more emotionally expressive (Fischer & LaFrance, 2015). This may indicate that it is more difficult for men to express and handle feelings caused by the illness. Men might experience more emotional challenges related to working and the redistribution of household responsibilities. If a man has previously been the breadwinner or has had certain responsibilities within the family, changing roles can be troublesome. The spouse's understanding and knowledge of the disease will have a major impact on the situation. Family members can also have problems adapting to a new role as a supporter (Commodore‐Mensah & Dennison Himmelfarb, 2012.) The responsibility of taking care of the person with CAD can cause stress (Andersson et al., 2013; Jackson et al., 2012). Family members are forced to take more responsibilities in daily life, and this can affect their own well‐being and influence their ability to offer support in the rehabilitation process (Jackson et al., 2012; Koerich, Baggio, Erdmann, Lanzoni, & Higashi, 2013).

An interesting observation was that having children in the family was significantly associated with issues encumbering rehabilitation. In this study, it was necessary to combine categories to enable logistic regression analysis, so it was not distinguished whether there were underage or adult children in the family. Concerns for children and their well‐being through the illness of a parent can cause stress in the family. Andersson et al. (2013) point out that there may be concerns and worries in the family about how underage children cope with their grief, the impact a parent's illness has on them and how they should be supported.

In this study, respondents who lived alone had fewer encumbering issues in rehabilitation. It might be that, while living alone, disagreements and other challenges do not severely strain family relationships. A person diagnosed with CAD needs information and support to cope with the illness, but it is not an absolute that optimum support can only be received from family members living in the same household. However, an essential consideration is that men who live alone do have greater myocardial infarction fatality (Kilpi et al., 2015), as there is a risk that a person who lives alone does not necessarily get help early enough.

An interesting finding in this study was that living with grown children was related to having more encumbering issues in the family. This might be due to various reasons relating to the child, the parent or the family situation. Grown children might have some socio‐economic challenges, which increases the likelihood of co‐residence (Isengard & Szydlik, 2012). An earlier study dealing with social support given by family members showed that children are a statistically significant source of support for persons diagnosed with CAD (Roos et al., 2012), but studies concerning the support given by grown children are rare.

### 4.1 Strengths and limitations

In this study, most respondents (76%) were men. However, this corresponds fairly well to the gender distribution of CAD patients in Finland (The Social Insurance Institution of Finland, 2019). Men and women may experience interpersonal relationships in different ways; consequently, with more women respondents, we could have achieved different results. Additionally, the data were collected from one university hospital, which is part of a big hospital district responsible for the care of 900,000 people, so the results represent the Finnish population quite well. Although the study data were collected based on a power analysis, some subgroups remained quite small such as the number of respondents living with underage children (*N* = 11) and respondents living with adult children (*N* = 11). Because of this, it is necessary to be cautious about the generalizability of the results. The time of the CAD diagnosis and the respondents' treatment were not limited in any way. The respondents in this study were all patients with CAD, but they were in quite different situations; some of them had been diagnosed with CAD many years ago, but for other respondents, adjusting to the illness was new. The severity of the illness and treatments also varied among participants. Almost all respondents had received thrombolytic therapy, one in ten were treated with coronary artery bypass grafting and three‐quarters had had PCI. In a registry study of infarction patients, approximately 37% per cent of patients were treated with PCI and about 7% with bypass surgery (Kyto et al., 2019). This supports the representativeness of the data in relation to Finnish CAD patients.

One factor that might have caused bias is that patients whose condition was weak and who needed further hospital treatment were excluded from the study. It can be stated that the sample was wide‐ranging and gives a diverse view of the population of interest. It was not possible to conduct a proper non‐response analysis because of the lack of information about the persons who declined to participate. Based on the pilot study (Tuomisto et al., 2014), the length of the questionnaire can be considered appropriate, as the questionnaires were filled out conscientiously and there was only one proposal for improvement (clarification of a single wording). The average time taken to complete the survey was 25 min. Also, the high response rate of this study supports the good suitability of the FIRE scale for people with CAD. Considering the FIRE scale, it should be considered as a limitation that the acceptable cut‐off values for analysis of subscales have not been determined and the instrument has not yet been used in other studies besides this research project, which can have an impact on the generalization, validity and replication of the study.

## 5 CONCLUSIONS

This study adds to our knowledge of the self‐managed rehabilitation phase at home and of the families' involvement from the point of view of persons with CAD. Based on these results, the following suggestions are given for the nursing practice. Patients and their family members, supported by healthcare professionals, evaluate the current family situation and living conditions and express their thoughts and emotions related to the illness. Patients and their families should receive appropriate information concerning the impact of family relations, emotional well‐being and supportive family during cardiac recovery. Especially, men and CAD patients living with grown children need nursing support and guidance for preventing issues encumbering rehabilitation in the family.

## CONFLICT OF INTEREST

The authors declare no conflict of interest.

## AUTHOR CONTRIBUTIONS

All listed authors met the authorship criteria and made substantial contributions to the conception and design or analysis and interpretation of data, drafting the article or revising it critically for important intellectual content and the final approval of the version to be published.
